# Artificial womb technology and clinical translation: Innovative treatment or medical research?

**DOI:** 10.1111/bioe.12701

**Published:** 2019-11-29

**Authors:** Elizabeth Chloe Romanis

**Affiliations:** ^1^ Centre for Social Ethics and Policy Department of Law University of Manchester Manchester United Kingdom of Great Britain and Northern Ireland

**Keywords:** artificial wombs, innovative treatment, medical research, neonatal intensive care, partial ectogenesis, research ethics

## Abstract

In 2017 and 2019, two research teams claimed ‘proof of principle’ for artificial womb technology (AWT). AWT has long been a subject of speculation in bioethical literature, with broad consensus that it is a welcome development. Despite this, little attention is afforded to more immediate ethical problems in the development of AWT, particularly as an alternative to neonatal intensive care. To start this conversation, I consider whether experimental AWT is innovative treatment or medical research. The research–treatment distinction, pervasive in regulation worldwide, is intended to isolate research activities and subject them to a greater degree of oversight. I argue that there is a tendency in the literature to conceptualize AWT for partial ectogenesis as innovative treatment. However, there are sufficiently serious ethical concerns with experimental AWT that mean that it must not be first used on humans on the basis that it is a ‘beneficial treatment’. First, I outline the prospects for translation of AWT animal studies into treatment for human preterms. Second, I challenge the conceptualizations of experimental AWT as innovative treatment. It must be considered medical research to reflect the investigatory nature of the process and guarantee sufficient protections for subjects. Identifying that AWT *is* research is crucial in formulating further ethico‐legal questions regarding the experimental use of AWT. Third, I demonstrate that clinical trials will be a necessary part of the clinical translation of AWT because of requirements laid out by regulators. I consider the justification for clinical trials and highlight some of the crucial ethical questions about the conditions under which they should proceed.

## 1 INTRODUCTION

Two research teams, in the U.S. and Australia, claim to have established proof of principle for artificial womb technology (AWT).1Partridge, E. A., Davey, M. G., Hornick, M. A., McGovern, P. E., Mejaddam, A. Y., Vrecenak J. D… Flake, A. W. (2017). An extra‐uterine system to physiologically support the extreme premature lamb. *Nature Communications, 8*, 1–15; Usuda, H., Watanabe, S., Saito, M., Sato, S., Musk, G., Fee, E… Kemp, M. W. (2019). Successful use of an artificial placenta to support extremely preterm ovine fetuses at the border of viability. *American Journal of Obstetrics and Gynecology, 221*(69), e1–17. Both teams have designed ‘artificial womb devices’ that they claim will revolutionize the treatment of preterm neonates. The devices, the EVE2Ex‐vivo environment. platform and the biobag, are intended to facilitate the process of gestation ex utero (ectogenesis), enabling preterms to continue to develop. When artificial wombs (AWs) are used to *continue* gestation ex utero this is described as *partial* ectogenesis.3Kaczor, C. (2005). *The edge of life: Human dignity and contemporary bioethics, philosophy and medicine.* Dordrecht, the Netherlands: Springer, p. 113. Partial ectogenesis could significantly reduce mortality and morbidity rates amongst preterms. Both teams explicitly anticipate the clinical application of their device as a treatment for preterms in the near future. Little attention has been afforded, however, to how experimental AWs might be ethically justified as an alternative to existing treatment.

Following recent animal studies of the biobag (2017) and EVE platform (2019), there has been renewed interest in the socio‐ethical implications of AWs.4Cohen, I. G. (2017). Artificial wombs and abortion rights. *Hastings Center Report 47 (inside back cover).*
 This debate treats the development and human use of AWs as a foregone conclusion. The focus on future implications neglects more immediate ethical problems concerning the translation of AWT from concept and animal studies into treatment. Arguments are frequently made that ectogenesis is a moral imperative5Smajdor, A. (2007). The moral imperative for ectogenesis. *Cambridge Quarterly of Healthcare Ethics, 16*(3), 336–345. and a welcome development.6Kendal, E. (2017). The perfect womb: Promoting equality of (fetal) opportunity. *Journal of Bioethical Inquiry 14*(2), 185–194, pp. 185–186. Claims that AWT should be actively sought imply that risks in research are justified to achieve this end. Often scholars ignore the fact that research on humans, both pregnant people and preterms, would be necessary, or they downplay the risks involved. Work exploring the ethics of experimental AWT focuses on full ectogenesis (rather than partial ectogenesis)7Raskin, J. M., & Mazor, N. (2006). The artificial womb and human subject research. In S. Gelfand & J. R Shook, J. R. (Eds.), *Ectogenesis: Artificial womb technology and the future of human reproduction* (pp*.* 159–182). New York, NY: Rodopi; Alghrani, A. (2018). *Regulating assisted reproductive technologies*. Cambridge, U.K.: Cambridge University Press. or pre‐dates the recent scientific studies8Alghrani, A., & Brazier, M. (2011). What is it? Whose it? Re‐positioning the fetus in the context of research? *Cambridge Law Journal, 70*(1), 51–82. and is therefore more abstract. This paper addresses this gap in the literature. How and when might it be ethical to use experimental AWT on humans? These questions are increasingly important as researchers consider translating their devices into treatment for preterms.

In this paper I address these questions by considering whether AWT is an innovative treatment or medical research. A distinction between innovative treatment and research is pervasive in bioethical literature,9Beauchamp, T. L., & Saghai, Y. (2012). The historical foundations of the research–practice distinction in bioethics. *Theoretical Medicine and Bioethics, 33*(1), 45–56, p.46. The research–innovative treatment distinction has been criticized: Kass, N. E., Faden, R. R., Goodman, S. N., Pronovost, P., Tunis, S., & Beauchamp, T. (2013). The research–treatment distinction: A problematic approach for determining which activities should have ethical oversight. *Hastings Center Report, 43*(1), S4–S15. However, the distinction remains widely accepted in the literature, and is embedded in law in the U.S.A., U.K. and Australia. and is at the foundation of regulation in many jurisdictions.10For example the U.S.A.: The National Commission for the Protection of Human Subjects of Biomedical and Behavioural Research. (1979). *The Belmont report: Ethical principles and guidelines for the protection of human subjects of research.* Retrieved from https://www.hhs.gov/ohrp/sites/default/files/the-belmont-report-508c_FINAL.pdf and the U.K.: *Simms v Simms* [2003] 1 Fam 83. The basis of this distinction is that research subjects need more protection than patients receiving medical treatment. Research is directed towards the production of generalizable knowledge in the interests of future patients/medical science.11Lewens, T. (2006). Distinguishing treatment from research: A functional approach. *Journal of Medical Ethics 32*(7), 424–429, p. 425. Innovative treatment, however, aims to treat the patient in their best interests. The subject of medical research is more vulnerable than a patient receiving medical treatment because there is not the same guarantee that the investigator is acting in their interests.12Menikoff, J. (2006) *What the doctor didn’t say: The hidden truth about medical research.* New York, NY: Oxford University Press, pp. 17–19. Consequently, research is subject to a higher level of ethical oversight13Berkman, B. E., Hull, S. C., & Eckstein, L. (2014). The unintended implications of blurring the line between research and clinical care in a genomic age. *Personalized Medicine, 11*(3), 285–295, p. 286. than the normal ethical constraints of medical practice (treatment in the patient’s best interests)14Chan, S. (2018). Research translation and emerging health technologies: Synthetic biology and beyond. *Health Care Analysis, 26*(4), 310–325, p. 319. to protect the research subject. Because of the ethical tensions innate in the innovative treatment–research distinction, categorizing activities is essential wherever possible. Berkman et al. advocate that this is the easiest way to ensure that activities are afforded the appropriate ethical oversight and that investigators/clinicians are aware of their responsibilities to subjects/patients.15Berkman et al., op. cit. note 13, p. 287.


It is often assumed that AWs are an extension of (but an improvement on) current methods in neonatal intensive care (NIC).16For example, Singer, P., & Wells, D., (2006). Ectogenesis. In S. Gelfand & J. R Shook (Eds.), *Ectogenesis: Artificial womb technology and the future of human reproduction* (pp*.* 9–25). New York, NY: Rodopi, p. 10. The assumption implies that partial ectogenesis should be conceptualized as innovative medical treatment. The danger with such an approach, however, is that it enables experimental AWs to be utilized on an ad hoc basis without objective ethical scrutiny. By failing to recognize the difference between AWT and NIC, we potentially expose pregnant people and preterms to harm. In this paper, I argue that AWT must be conceptualized as medical research, and be subject to the same strict criteria as all research. This paper does not examine all the ethical constraints that should regulate experimental AWT, but instead establishes that there are sufficiently serious ethical concerns with experimental AWT that means it must not be first used on humans on the basis that it is a ‘beneficial treatment’.

First, I outline the prospects for experimental AWT with human subjects. Second, I review how AWT has been conceptualized in the bioethical literature, demonstrating why these conceptualizations are problematic. I argue that experimental AWT must be considered medical research to better reflect the investigatory nature of the process, and to better guarantee protections for the subjects of experimental AWT. I outline that AWT would be required by regulatory authorities to be the subject of extensive clinical trials before such devices are approved for more general use. Third, I consider whether and how such clinical trials should be conducted by highlighting some of the critical ethical questions that must be addressed in designing such trials.

## 2 THE PROSPECTS FOR ARTIFICIAL WOMB TECHNOLOGY

In 2017, Partridge et al. published the results of their artificial womb‐like device (the biobag) designed to continue gestation ex utero. Catheters imitate umbilical cord access and facilitate water and nutrient provision and waste product removal. An oxygenator ensures oxygen provision but allows the subject’s heartbeat to control circulation as in utero. The subject is sealed in (synthetic) amniotic fluid, facilitating sustenance delivery and protecting it from infection.17Partridge et al., op. cit. note 1, pp. 2–4. The biobag was able to sustain preterm lamb foetuses, developmentally equivalent to ‘just‐viable’ human preterms, for 28 days. 100% of the biobag subjects survived and were successfully ‘delivered’. All research subjects appeared healthy and to have developed (evidencing successfully continued gestation). The biobag researchers acknowledge that the device needs further refinement,18Ibid: p. 10. and their results need additional validation. They conclude, however, that their device might soon be ready for human testing, identify their potential clinical target population, and comment on the justification for use of experimental AWT on humans.19Ibid: p. 11.


In 2019, Usuda et al. published the second trial of the EVE platform.20Usuda et al., op. cit. note 1. The EVE device has a similar design, sealing the subject in a warm amniotic fluid bath in a sterilized plastic bag. The subject’s heartbeat, an oxygenator and catheters maintain circulation. In a 2017 study,21Usuda, H., Watanabe, S., Miura, Y., Saito, M., Musk, G. C., Rittenschober‐Böhm, J… Kemp, M. (2017). Successful maintenance of key physiological parameters in preterm lambs treated with ex vivo uterine environment therapy for a period of 1 week. *American Journal of Obstetrics and Gynecology, 217*(457), e1–13. the EVE platform sustained lamb foetuses for a shorter period than the biobag study, and had a higher incidence of morbidity and mortality. The authors were reserved about the potential clinical application of EVE therapy and directed their conclusions towards redesign. In 2019, they published the results of a study using their redesign. Usuda et al. reported an improved survival rate, and posited that, although preliminary, their findings ‘demonstrate the potential clinical utility of a further refined EVE therapy system to improve outcomes for extremely preterm infants’.22Usuda, et al., op. cit. note 1, p. e2.


These studies are encouraging but have limitations. The devices have only been tested on small sample sizes for short durations. Further validation of results is necessary in repeated, longer studies. Moreover, the outcome of these studies should not be considered sufficiently promising to allow use on human subjects without significant refinement. The EVE study had a survival rate of 87.5%, there was an incidence of brain damage, and several subjects displayed early signs of liver dysfunction. These risks *may* be no worse than those that routinely occur in NIC. It is important, however, that the specific risks and uncertainties are acknowledged. Finally, lambs have a different physiology from humans. Thus, the devices may be less successful when used on humans. Testing on animals, such as primates, with physiologies more similar to humans, is necessary to better understand the likelihood of AWs gestating humans. Significant hurdles must be overcome before AWs are ready for human testing. It is clear, however, that researchers believe that their preliminary results support the expectation that their devices could prolong human gestation ex utero. The process of refinement and validation may not be lengthy, as development in this area is fast‐paced. The EVE team produced a redesign in just two years. There are strong incentives driving this research—researchers are primarily concerned with improving outcomes for preterms and their parents, but are also intrigued by the bioengineering problems and the possible prestige of developing the first ‘artificial womb’—and there is available funding.23Woolfrey, J. (2006). Ectogenesis: Liberation, technological tyranny or just more of the same? In S. Gelfand & J. R Shook (Eds.), *Ectogenesis: Artificial womb technology and the future of human reproduction* (pp*.* 77–89). New York, NY: Rodopi, pp. 130–131. In an interview, Dr Flake (biobag study lead) alluded to a future ‘a decade from now’ when preterms are treated with AWT rather than conventional NIC,24Children’s Hospital of Philadelphia. (2017). *Unique womb‐like device could reduce mortality and disability for extremely premature babies.* Retrieved from https://medicalxpress.com/news/2017-04-unique-womb-like-device-mortality-disability.html?utm_source=TrendMD%26utm_medium=cpc%26utm_campaign=MedicalXpress_TrendMD_1. suggesting we are on the cusp of the technology. It is important that we start considering some of the ethico‐legal issues inherent to experimental AWT before they are considered a clinical option.

Identifying how AWT might be translated into a clinically effective device is essential to addressing any ethical issues in the process. Both research teams declare that the objective of their device is to provide human preterms with more comprehensive support and to improve clinical outcomes. The studies, however, might potentially differ in their approach to future clinical translation. There is a presumption in the literature that AWT will be used beyond current conceptions of viability.25Alghrani and Brazier focus on technology designed to lower the viability threshold, suggesting that they envisaged AWT would be trialled in this way: Alghrani & Brazier, op. cit. note 8, p. 53. The bioethical literature focuses on ectogenesis collapsing the viability threshold: Cohen, op. cit. note 4. This is dependent on AWT being used before 24 weeks. This is not an explicit claim that AWT should be initially trialled like this, but does envisage experimental use of AWT below the viability threshold at some point. The biobag study, however, identifies their clinical population as the ‘just‐viable’ preterm (23–25 weeks) and is explicit that the authors have no intention of challenging the viability timeline. Their aim is only to reduce the incidence of mortality and morbidity amongst those preterms that we already know have some capacity to survive.26Partridge et al., op. cit. note 1, p. 11. In this approach to clinical translation, experimental AWT would involve using a method that is conceptually sound but unproven in humans, in place of the existing approach (conventional NIC) that has mixed results. Conventional NIC routinely ensures the survival of preterms.27Azad, K., & Matthews, J. (2016). Preventing newborn deaths due to prematurity. *Best Practice & Research: Clinical Obstetrics and Gynaecology, 36*, 131–144, p. 132. Before 26 weeks, however, preterms remain unlikely to survive the common complications associated with prematurity and resulting from NIC.28Lissauer, T., & Clayden, G. (eds.) (2012). *Illustrated textbook of pediatrics.* London, U.K.: Mosby Elsevier, p. 159. Only approximately 9% of preterms born at 22 weeks survive.29Stoll, B. J., Hansen, N. I., Bell, E. F., Walsh, M. C., Carlo, W. A., Shankaran, S… Higgins, R. D. (2015). Trends in care practices, morbidity and mortality of extremely preterm neonates, 1993–2012. *Journal of the American Medical Association, 314*(10), 1039–1051, p. 1045. One study reports that the survival rate increases to 33% at 23 weeks, and to 65% at 24 weeks.30Ibid: p. 1045. Complications in NIC include lung damage caused by ventilation, cardiac failure, and infection.31Lissauer & Clayden, op. cit. note 28, p. 159. Of preterms born at 26 weeks that do survive, 50% have a severe impairment following complications.32Ibid: p. 159. This increases to 75% amongst preterms at 23 weeks.33Moore, T., Hennessy, E. M., Myles, J., Johnson, S. J., Draper, E. S., Costeloe, K. L., & Marlow, N. (2012). Neurological and developmental outcome in extremely preterm children born in England in 1995 and 2006: The EPICure Studies. *British Medical Journal*, *345*, e7961. Despite overall mortality rates improving in NIC over time, outcomes for extremely preterm infants (< 28 weeks) have not meaningfully changed in the last two decades.34Stoll, B.J. et al, op. cit. note 29, p. 1045. Glass, H. C., Costarino, A. T., Stayer, S. A., Brett, C., Cladis, F., & Davis, P. J. (2015) Outcomes for extremely premature infants. *Anesthesia & Analgesia, 120*(6), 1337–1351. Moreover, conventional care does not consistently produce good outcomes, because individual circumstances vary greatly, and thus it is hard to predict the outcome in any individual case.35All the data we have about the likelihood of premature infants surviving (based on factors such as gestational age and weight) are prediction models involving a multitude of factors. Kipnis argues that whilst there may be generalized data to support the likelihood of a good outcome (assuming a certain weight and gestational age), NIC is a unique situation. In providing treatment intended to result in the best outcome (survival with limited deficits), it is also then inevitable that the likelihood of the worst outcome (death after suffering or survival with intolerable deficits) simultaneously increases. Kipnis, K. (2007). Harm and uncertainty in newborn intensive care. *Theoretical Medicine and Bioethics, 28*, 393–412, p. 404. There is also uncertainty in every individual case, because we cannot accurately predict the incidence of every complication, from how developed a particular subject’s lungs are to occurrence of necrotizing encephalitis.


The EVE authors make no explicit attempts to identify their clinical population, but note that they envisage the EVE platform aiding preterms ‘close to or at the border of viability’.36Usuda et al., op. cit. note 1, p. e2. The study emphasizes that the lamb subjects on which the device was tested were chosen so that they as far as possible, ‘approximated the size and weight of a human fetus close to the border of viability (21–24 weeks of gestation).’37Ibid. This implies that the authors anticipate the experimental use of EVE on preterms not yet considered viable (just below that threshold). This potentially presents an alternative clinical translation strategy of initially trialling AWT on preterms that are delivered alive but are so functionally immature that resuscitation would not typically be attempted (<** **22 weeks).38Nuffield Council on Bioethics. (2006). *Critical care decisions in fetal and neonatal medicine: Ethical issues.* Plymouth, U.K.: Latimer Trend & Company, p. 21. These two approaches (using viable, or not yet viable preterms) invoke different ethical issues.

## 3 EXPERIMENTAL AWT IS MEDICAL RESEARCH

The conventional account of partial ectogenesis is that it is already a ‘partial reality’,39Singer & Wells, op. cit. note 16, p. 11. and full ectogenesis will come about in the natural evolution of NIC.40Alghrani, A. (2007). The legal and ethical ramifications of ectogenesis. *Asian Journal of WTO & International Health Law and Policy, 2*(1), 189–212, p. 193. Singer and Wells argued in 1984 that the ability of modern medicine to ensure the survival of preterms meant that ectogenesis was already a partial reality.41Singer & Wells, op. cit. note 16, p. 10. Both Cannold42Cannold, L. (2006). Women, ectogenesis and ethical theory. In S. Gelfand & J. R Shook (Eds.), *Ectogenesis: Artificial womb technology and the future of human reproduction* (pp*.* 47–58). New York, NY: Rodopi, p. 47. and Alghrani43Alghrani op. cit. note 7, p. 146. refer to partial ectogenesis as a reality demonstrated by the ‘gestation of premature babies in incubators’.44Cannold, op. cit. note 42, p. 47. Even those who do not expressly claim that partial ectogenesis is already facilitated by incubators often imply that AWs would be only a slight ‘advancement of’ NIC45Steiger, E. (2010). Not of woman born: How ectogenesis will change the way we view viability, birth and the status of the unborn. *Journal of Law and Health, 23*(2), 143–171, p. 150.—a case of improving current technology to enable it to support younger preterms.46Alghrani, op. cit. note 40, p. 191. Coleman suggests that ‘if premature new‐borns are saved earlier and earlier stages of gestation then eventually… ectogenesis may be discovered by default’.47Coleman, S. (2004). *The ethics of artificial uteruses: Implications for reproduction and abortion.* Aldershot, U.K.: Ashgate, p. 45. Singer and Wells posited that ectogenesis would be further developed ‘by accident’, not because of ‘researchers deliberately seeking to make ectogenesis possible but rather [as a result of] doctors attempting to save… premature babies’.48Singer & Wells, op. cit*.* note 16, p. 10. Alghrani suggests that efforts to perfect NIC mean that ‘ectogenesis may not be as far off as… imagined’.49Alghrani, A. (2008). Regulating the reproductive revolution: Ectogenesis – a regulatory minefield. In M. Freeman (Ed.), *Law and Bioethics* (pp. 303–332). Oxford, U.K.: Oxford University Press, p. 303.


These conceptualizations of AWT as similar to existing routine treatment are an attempt to contextualize claims about the implications of AWs by ‘demonstrating’ that AWT is a ‘real, serious technology and thus something worthy of ethical debate’.50Hedgecoe, A. (2010). Bioethics and the reinforcement of socio‐technical expectations. *Social Studies of Science, 40*(2), 163–186, p. 172. AWT is ‘already here’, so ectogenesis is merely trying more of the same type of intervention in preterms born earlier. Singer and Wells assert that, as an extension of current methods, AWT would not be ‘potentially reckless with human life’.51Singer & Wells, op. cit*.* note 16, p. 16. They envisage AWT as technology that builds on already established medical approaches with therapeutic intention in each use. This characterization of AWT may not have been intended as a commentary on the status of these technologies as innovative treatments rather than research; however, this is the implication of conceiving of AWT as merely an extension of NIC. This characterization misleadingly implies that AWT is a beneficial treatment rather than a novel process embroiled in uncertainty. The conceptualization of AWT as already a partial reality, or as a development of an already established technological approach, is flawed for several reasons. These reasons illustrate that the clinical translation of AWT is research.

First, underlying the supposition that partial ectogenesis is already a partial reality is the flawed assumption that incubation and gestation are conceptually identical. I have argued that incubation is a bid to ‘rescue’ a preterm by assisting that preterm with life functions it is attempting, or beginning to attempt, to sustain itself independently. The process of gestation, however, is different; it is the process of the creation of humans.52Romanis, E. C. (2018). Artificial womb technology and the frontiers of human reproduction: Conceptual differences and potential implications. *Journal of Medical Ethics*, *44*, 751–755, p. 754. AWT marks a complete shift in approach to the treatment of underdeveloped human preterms: from ‘rescuing’ a preterm by intervening to assist in the performance of life functions necessary for independent life, to facilitating the ‘creation’ of the subject by continuing the process of gestation ex utero. Conventional NIC requires that preterms withstand ventilation53Hendricks, J. (2012). Not of woman born: A scientific fantasy. *Case Western Reserve Law Review, 62*, 399–445, p. 405. because oxygen must be obtained through use of their lungs (pulmonary gas exchange). Conventional incubators, therefore, will never be capable of facilitating ectogenesis even as they improve because, in requiring a preterm to use their lungs, the continued development of the lungs is precluded.54Romanis, op. cit. note 52, p. 753. AWT devices are purposefully designed to avoid the subject having to utilize their lungs. Instead, gas exchange is performed using a catheter imitating gas exchange through the placenta in utero.55Usuda et al., op. cit. note 1, p. e2. The intention is to try to build an environment in which a subject can behave more like a ‘foetus’ than a ‘baby’. AWT is an attempt to artificially replicate and replace the biological process of gestation and to facilitate the existence of humans ex utero that do not need to exercise their life functions to survive. The important point in raising this distinction is to highlight that experimental AWT is an advancement of NIC only in the sense that it seems conceptually superior (if successful) in process and outcomes. Furthermore, attempting to use AWT on preterms means completely abandoning *all* previously established, and proven, treatment methods on a vulnerable population to test an approach with unknown short‐ and long‐term consequences, thus exposing the subject to risks. Conventional NIC is certainly not risk‐free. However, we know what those risks are and how we can attempt to mitigate them (even if we cannot be certain that this will work); we also anticipate that after 26 weeks there is a reasonable chance that preterms will survive treatment.56Lissauer & Clayden, op. cit. note 28, p. 159. Experimental AWT could involve unknown risks and potentially result in subjects experiencing worse outcomes than they might have done if treated by conventional methods. Experimental AWT, therefore, needs justification.

Experimental AWT is embroiled in more uncertainty than conventional NIC. Novel approaches sometimes constitute innovative treatments where there is some clinical basis for believing that the patient will experience a direct benefit.57Levine, J. (1979). The boundaries between biomedical or behavioral research and the accepted and routine practice of medicine. In National Commission for the Protection of Human Subjects of Biomedical and Behavioral Research. *Appendix to the Belmont Report: Volume I* (pp. 1–44). Retrieved from https://videocast.nih.gov/pdf/ohrp_appendix_belmont_report_vol_1.pdf, p. 34. If there were a reasonable expectation that experimental AWT would directly benefit individual preterms, it would not need to be considered research. If ‘just viable’ preterms were used to trial AWT, it could be argued that there is already evidence that these ‘just‐viable preterms’ are capable of surviving with treatment; therefore, there is a valid clinical basis for treating them with AWT. However, knowing that there are instances in which this group can survive with support cannot be equated to a reasonable expectation that they will benefit from *AWT.* In this context, benefit would (or should) mean that the preterm experiences a better outcome than that anticipated from NIC. The approach envisaged by AWT (continuing gestation) is conceptually sound. However, there is uncertainty in translating that theoretical approach into reality. There is preliminary data suggesting that ex utero gestation is possible for periods with animals. Current animal data, however, are not sufficient to demonstrate a reasonable expectation of clinical benefit because of the substantial differences in physiology. This is why standard research pathways involve human trials after animal testing before approval. Until it is tried for the first time, there is no *clinical* evidence supporting experimental AWT in humans. The lack of clinical validity gives us good reason to presume that AWT should be considered research.

Second, in research the investigator primarily intends to produce generalizable knowledge,58National Commission for the Protection of Human Subjects of Biomedical and Behavioural Research, op. cit. note 12, pp. 2–3; Department of Health. (2005). *Research governance framework for health and social care.* London, U.K.: Central Office of Information, p. 3. whereas when providing innovative treatment the intention is to benefit the individual patient.59Brody, H., & Miller, F. G. (2013). The research–clinical practice distinction, learning health systems, and relationships. *Hastings Center Report, 43*(5), 41–47, p. 44. Where generalizable knowledge is the primary goal, objective scrutiny is necessary to ensure that health is not ‘unduly sacrificed in experimental design’.60Lewens, op. cit. note 11, p. 425. Dr Flake envisages that AWT ‘could establish a new standard of care for this subset of extremely premature infants’.61Children’s Hospital of Philadelphia, op. cit. note 24. The EVE team expressly state that their objective is to develop ‘a new therapeutic option’ replacing conventional ventilation.62Usuda et al., op. cit. note 1, p. e2. The researchers clearly perceive their devices as different from traditional approaches and hope that their research will improve outcomes for preterms. The objective in developing AWT is that eventually, if the devices work as well as anticipated, AWT could more reliably support human preterms and reduce the incidence of mortality and morbidity. Ultimately, if researchers can prove the validity of their approach, AWT may replace conventional NIC (and associated complications). To do this, evidence is needed to establish that AWs can consistently achieve better outcomes. Thus, the primary objective of experimental AWT is the production of generalizable knowledge establishing that AWT is a replacement for conventional technologies.

This objective does not preclude the possibility that physician‐investigators hope or intend that experimental AWT will benefit individual subjects of the device, perhaps by ensuring their survival where it was uncertain or by reducing the incidence of complications. This intention is, however, secondary to the production of generalizable knowledge. Initial clinical translation will involve trial and error. If researchers intended to provide the best treatment available to a preterm (assuming that the preterm was ‘just viable’ at 23–25 weeks), the reasonable course of action would be to provide conventional therapies known to have some success rather than to trial a device with uncertain outcomes as an alternative. For example, survival (irrespective of morbidity) rates of NIC at 25 weeks gestation are around 81%.63Stoll et al, op. cit. note 29, p. 1045. It might be, prima facie, more justifiable to trial the technology on preterms at the threshold of this category, for example 22 weeks, where NIC is a much less reliable option. In any case, experimental AWT involves uncertainty and substantial risks. Attempting to assist any individual preterm is, consequently, not the primary motivation of experimental AWT. Even if AWT resulted in benefit for individual subjects in every case, this does not render the procedure treatment rather than research.64Dickens, B. M. (1975). What is a medical experiment? *Canadian Medical Association Journal, 113*(7), 635–639, p. 635.


Finally, the substantial risks assumed by the subject of experimental AWT are unlikely to be justifiable *on the basis that* the individual subject will benefit. Some risk of harm is innate in all medical practice (including innovative treatment) and is considered justifiable where the patient stands to benefit from that treatment.65Berkman et al. op. cit. note 13, p. 286. Because research is not primarily aimed at securing a patient’s medical best interests, the justification offered is consequentialist: that any risk tolerated in the experiment might be justifiable based on contribution to knowledge that will have further good outcomes, such as improving the prospects for future patients.66Brody & Miller, op. cit. note 59, p. 44. How risks of treatment are justified is therefore useful in distinguishing between innovative treatment and research. Substantial risks are involved in experimental AWT, such as cardiac failure from circuit overload, liver dysfunction because of improper nutrient supply, or brain injury. Circuit malfunction is also a significant risk because there is a ‘delicate balance between adequate and excessive circuit flow’.67Partridge et al., op. cit. note 1, p. 10. Animal studies are unhelpful here because circuit flow/nutrition will be impacted by the size of the subject. The initial use of experimental AWT on humans may not necessarily benefit the first subjects but could benefit preterms in the future.68Alghrani, op. cit. note 7, p. 135. Without a reasonable expectation of success, and with an understanding of the substantial risks involved, AWT should not be considered justifiable because it is in any individual preterm’s interests. It could, however, be considered justifiable because of the potential to revolutionize the future of preterm care.

Research on developing human beings is controversial, especially when of uncertain benefit. The World Medical Association,69World Medical Association. (2000). *Ethical principles for medical research involving human subjects.* Retrieved from https://www.wma.net/policies-post/wma-declaration-of-helsinki-ethical-principles-for-medical-research-involving-human-subjects/. British Medical Research Council70Medical Research Council. (2004). *Medical research involving children.* Retrieved from https://mrc.ukri.org/documents/pdf/medical-research-involving-children/. and U.S. Department for Health and Human Services,71US Department for Health and Human Services. (2018). *DHHS regulations.* Retrieved from https://www.ecfr.gov/cgi-bin/retrieveECFR?gp=%26SID=83cd09e1c0f5c6937cd9d7513160fc3f%26pitd=20180719%26n=pt45.1.46%26r=PART%26ty=HTML#se45.1.46_1205. however, all accept that non‐therapeutic research in these circumstances can be justifiable where it is intended to benefit future preterms, and can only be conducted on the identified population.72Mason, J. K., & Laurie G. T. (2013) *Mason & McCall Smith’s law & medical ethics*. Oxford, U.K.: Oxford University Press, pp. 650–651, 687. Brazier and Alghrani argue that the potential of AWT to ‘improve the care of premature babies provides a strong case for permitting ethically approved research’.73Alghrani & Brazier, op. cit. note 8, p. 71. Thus, experimental AWT can be justified, although the reality, according to Brazier and Alghrani, is that research into ectogenesis being in any initial subject’s best interests is contestable.74Ibid: p. 70. Thus, AWT is research. The functional importance of conceptualizing experimental AWT in this way is that research involves formal protocol, robust study design, and procedures designed to ensure the protection of subjects and to ensure the production of generalizable knowledge.75Brody & Miller, op. cit. note 59, p. 45. If this research were to go ahead, it would first be in a clinical trial. Regulatory agencies, such as the Food and Drug Administration (FDA) in the United States or the Therapeutic Goods Administration (TGA) in Australia (where this technology is currently being developed) would require extensive clinical evidence before these devices could be utilized outside controlled processes. The FDA regulates medical devices by a classification system. ‘High risk devices’ include implanted devices and devices that are ‘life‐supporting or life‐sustaining… [or that present] a potential unreasonable risk of illness or injury’.76The definition is found in the U.S. Code of Federal Regulations concerning Food and Drugs 21 CFR §860.3 (c) (3). High‐risk devices are known as Class III devices. AWT is likely to fall into this category. By design, it is an advanced life‐support system. It is not implanted into the subject, but arguably the fact that a subject is so encased and intimately connected to the device means that the degree of support it provides is similar to an implanted device. Moreover, the uncertainty in the outcome for any individual subject means that there is the potential for unreasonable risks of adverse effects. The FDA has the authority to require that high‐risk devices secure pre‐market approval,77Following the U.S. Federal Food, Drug and Cosmetic Act, 21 U.S.C. §360e (1976), Class III devices (high‐risk) are required to secure premarket approval (with only a very limited number of exceptions). This section details the necessary process, including the information that must be supplied in order to secure approval. Further details about the requirements are provided in the U.S. Premarket Approval of Medical Devices Process Code of Federal Regulations: Ibid, §814. so that they cannot be generally used before clinical investigations78There are other research designs that can demonstrate the clinical utility of a medical device. There is, however, a general consensus that clinical trials are the best method to ensure the validity and generalizability of results. The regulatory approval process for a new medical device in multiple jurisdictions is also written assuming that clinical trials are the appropriate clinical investigation in the case of high‐risk devices. have demonstrated their safety and effectiveness.79The U.S. Federal Food, Drug and Cosmetic Act, op. cit. note 77, §360e (c) (1) (a) specifies that any application for premarket approval must include details about design, production, manufacturing and ‘full reports of all information… concerning investigations which have been made to show whether or not such device is safe and effective’. The FDA also has the authority to approve, require modifications to or disapprove all clinical trials before they begin.80Investigation cannot begin until approval from an IRB and FDA is secured: Premarket Approval of Medical Devices Regulations, op. cit. note 76, §812.42. The FDA can approve or disprove an application for investigations if there is reason to believe that risks do not outweigh the anticipated benefits for subjects or knowledge to be gained, there are issues with the investigational plan or methods, there is reason to believe that the device is/will be ineffective or informed consent is ineffective: §812.30. Studies with the potential for substantial risk must be approved both by an Institutional Review Board (IRB), and by the FDA.81U.S. Food & Drug Administration (2019) IDE Approval Process. Retrieved from https://www.fda.gov/medical-devices/device-advice-investigational-device-exemption-ide/ide-approval-process#sig_risk. Similar hierarchical classification systems, with processes requiring extensive evidence for devices considered higher risk, have been established in Australia82In Australia, medical devices are also classified on the basis of risk in s.41BD Therapeutic Goods Act 1989, and Regulation 3.2 Therapeutic Goods Regulations 2002. and Europe.83In the European Union, a classification system is established in Article XI Council Directive 93/42/EEC (1993). This classification system is implemented in the U.K. in the Medical Devices Regulations 2002, SI 2002/618. The Medicines and Healthcare Products Regulatory Agency has advised that EU directives governing the classification of new medical devices will remain in force, through the MDR 2002, immediately after any British exit from the EU. See: The Medical Devices (Amendment etc.) (EU Exit) Regulations 2019.


## 4 CLINICAL TRIALS AND AWT

It has been generally assumed that non‐beneficial research on developing human beings with the object of devising AWT could be ethically permissible.84Alghrani, op. cit. note 7, p. 135; Singer & Wells, op. cit. note 16, p. 16, and Alghrani & Brazier, op. cit. note 8, p. 78. Such conclusions, however, should be carefully drawn. In this section, I consider the justification for AWT research and posit that the consequentialist justification usually offered in the literature would be dependent on certain conditions. This investigation examines some of the questions that an IRB or the FDA might consider in deciding whether to approve an AWT clinical trial. The permissibility of these trials, I demonstrate, is embroiled in intricate ethical issues of research design. Before I engage in this discussion, I will briefly address the issue of the status of the subjects in a potential AWT trial.

I have argued elsewhere that the subject of an AW is a unique entity. A ‘gestateling’85A gestateling is ‘a human being in the process of ex utero gestation exercising, whether or not it is capable of doing so, no independent capacity for life’: Romanis, op. cit. note 52, p. 753. in an AW is not a neonate because it is not completely born. It is still undergoing the process of gestation (human creation), and is thus more ontologically similar in behaviour and physicality, in terms of exercising no capacity for independent life and having no interaction with others, to a foetus.86Romanis, E. C. (2019). Artificial womb technology and the significance of birth: Why gestatelings are not newborns (or fetuses). *Journal of Medical Ethics, 45*(11), 727–729, p. 728. It is also distinct from a foetus because it is not dependent on another person,87Ibid. a fact that will alter the way we treat it. There is, therefore, room to question whether a gestateling should be treated more like a foetus or a preterm. In the context of a potentially non‐beneficial research trial there are significant similarities in the way foetuses and preterms are treated, because regulators generally deem it impermissible to expose either to unnecessary risk and require researchers to maximize potential benefits and obtain parental consent. The U.S. Office for Human Research Protections (OHRP) has produced regulations on protecting human subjects in research88HHS Regulations 45 CFR 46 (2018). that provides guidance for IRBs making decisions. Subpart B provides additional protections for foetuses and neonates involved in research. §46.204 details the requirements in relation to foetuses and is explicit that any risk to the foetus must be solely by interventions that have some prospect of direct benefit, or if there is no direct benefit the risk must only be minimal and the purpose of research must be the generation of important biomedical knowledge that cannot be otherwise obtained. The informed consent of both parents must be obtained (so far as they are both available, and the consent of only the pregnant person is required if the research would also be for their benefit). §46.205 details the requirements for research on neonates of uncertain viability (likely to be those identified for any AWT study). This section requires that the research has the prospect of enhancing the probability of survival with the least possible risk and that the purpose of the research is important biomedical knowledge that cannot be obtained by other means with no added risk. The informed consent of either parent must be sought, unless the neonate is thought to be non‐viable in which case the consent of both parents is required (dependent on availability). We can see that there are significant similarities, therefore, in the practicalities of what IRBs will consider irrespective of whether subjects are deemed equivalent in moral status to foetuses or neonates. In this paper I will focus on the practicalities, in determining the permissibility of trials, that IRBs would consider important, for example what risks any subject is exposed to etc. because the approach taken would likely be similar irrespective of the subject’s moral status. The matter of how the moral status of the subject of an AW will determine its treatment in the future should be revisited.

Some might argue that AWT trials are ethically permissible because there is the potential that the subjects may benefit. It might be claimed that *some* chance of life (or a life without serious medical complications) is better than no chance at all. I have demonstrated that this justification is unlikely to be satisfactory because of uncertainty. I raise it again, however, to highlight that such a justification would be entirely dependent on identifying a particular category of research subject. There could be an identifiable population of subjects for whom the possible reward of ‘some chance at life’ could outweigh the chance of significant risks. These subjects are those who have little chance in NIC. In these cases, AWT could be considered at least as safe as NIC, and there would be no reason to suppose worse outcomes. The question would be instead how much potential suffering the subject is exposed to, and how much suffering is considered tolerable when weighed against a chance of life. Further, what gain in terms of ‘life’ is sufficient would need to be determined. For some parent(s), any additional time (even days) would be considered a benefit. This would, of course, be harder to justify if the subject were likely to experience heavy burdens in the study. It is difficult to support a claim that more mature preterms (> 24 weeks) benefit in any way by being subject to experimental AWT. Such a claim is dependent on whether researchers make a good case that AWT is as safe as, and potentially able to produce a better result than, current NIC. The benchmark is much higher for this group because they will have a chance of survival (> 50%) in NIC.89Glass et al. op. cit. note 34, p. 1338. Irrespective of what clinical population is identified, the only evidence researchers will have to attempt to justify an initial trial would be the results of animal studies. Lamb studies cannot provide sufficient data to demonstrate potential benefit in humans. Therefore, researchers may need to gather data by testing on more comparable animal models, such as primates, to justify a human trial in preterms90I am grateful to one of the anonymous reviewers for raising this point.—especially if they want to test the technology on subjects already considered viable. This will encompass its own ethical issues, but they are beyond the scope of this paper.

Any potential AWT trial may be high‐risk, but could potentially result in high rewards if the devices are proved successful. The general justification offered for these trials is the improved prospects for developing human beings and parent(s) in the future. Around 15 million babies are delivered preterm every year,91Lawn, J. E., Davidge, R., Paul, V. K., von Xylander, S., de Graft Johnson, J., Costello, A… Molyneux, L. (2013). Born too soon: Care for the preterm baby. *Reproductive Health, 10*(1), S1–S5. and prematurity remains the leading cause of neonatal death in high‐income economies.92Azad, K., & Matthews, J. (2016). Preventing newborn deaths due to prematurity. *Best Practice & Research Clinical Obstetrics & Gynaecology, 36*, 131–144, p. 132. Even though there has some increase in the number of preterms surviving post‐NIC, there has been no improvement for some time in the incidence of long‐term illness and complications affecting those who do survive.93Costeloe, K. L., Hennessy, E. M, Haider, S., Stacey, F., Marlow, N., & Draper, E. S. (2012) Short term outcomes after extreme preterm birth in England: Comparison of two birth cohorts in 1995 and 2006 (the EPICure studies), *British Medical Journal, 345*, e7976. NIC has some innate limitations, and we have potentially reached the limits of the capacities of these rescue technologies to aid preterms. AWT has also been emphasized as a potential aid to persons experiencing dangerous but wanted pregnancies.94Some have argued that it could potentially aid all women who want to reproduce but perceive pregnancy as too dangerous or want to avoid the risks associated with pregnancy: Smajdor, op. cit. note 5. Much good can be generated if AWT can save/improve the prospects for future preterms and persons experiencing (dangerous) pregnancy and limit the emotional distress experienced by parents. If we do not pursue AWT research, all of this potential could never be realized.95Alghrani, op. cit. note 7, p. 136. This consequentialist justification, frequently offered in the ethical literature,96Ibid: p. 135; Singer & Wells, op. cit. note 16, p. 16, and Alghrani & Brazier, op. cit. note 8, p. 78. should be approached with caution.

First, the logical corollary of a purely consequentialist justification is that, in order to maximize future good to future persons (especially on this scale), exposing research subjects to severe risks with little potential benefit is tolerable. Exposing subjects to considerable suffering, however, would not be compliant with regulatory requirements, which are a reflection of the ethical obligation not to abuse vulnerable populations. The OHRP Regulations, which contain requirements for IRBs making decisions about potential trials, do allow non‐beneficial research intended to result in important biomedical knowledge that cannot be obtained by other means, though subject to the caveat that there will be no added risk caused to the developing human being.97HHS Regulations, op. cit. note 88, §46.205. Second, a consequentialist justification is dependent upon research design, because if the correct research question is not identified and carefully answered (with appropriate methods), the future benefit is not possible. There are ethical issues embroiled in these methods that need to be addressed as part of any justification. Research subjects in any potential AWT trial would be vulnerable to exploitation because they are not participating in a wholly therapeutic arrangement. How can we ensure that a research trial is able to answer its research question? How do we mitigate concerns about coercion and parent(s) feeling pressured to consent to experimental procedures? How do we ensure that preterms are not exposed to unnecessary additional risk in the course of experimental procedures? These and other questions will require exploration in the process of ethical review before any research study is begun.

Clinical investigations are the only way that experimental AWT can result in generalizable knowledge. Emanuel et al. argue that to be ethical, research must be conducted in a methodologically rigorous manner: without ‘validity the research cannot generate the intended knowledge, cannot produce any benefit, and cannot justify exposing subjects to burdens or risks’.98Emanuel, E. J., Wendler, D., & Grady, C. (2000). What makes clinical research ethical? *Journal of the American Medical Association, 283*(20), 2701–2711, p. 2704. Reitsma and Moreno explain that doctors struggle to deny parents the newest available technology, regardless of questionable benefits and unknown risks, to facilitate parents’ wishes ‘not to give up’.99Reitsma, A., & Moreno, J. (2003). Maternal–fetal research and human research protections policy. *Clinics in Perinatology, 30*(1), 141–153, p. 144. If AWT were provided ad hoc to preterms in uncontrolled circumstances, generalizations about why and how AWT worked in some cases, and not in others, could not be made. Collating relevant information and accounting for confounding variables would be impossible. There would be no emerging evidence that could establish whether AWT is a viable alternative to (or replacement for) conventional NIC. Clinical trials ensure suitable research methods capable of producing generalizable knowledge. The Council of International Organisations of Medical Sciences advises that ‘scientifically unsound research on human subjects is ipso facto unethical in that it may expose subjects to risks…. to no purpose’.100Council for International Organizations of Medical Sciences. (1993). *International ethical guidelines for biomedical research involving human subjects.* Geneva, Switzerland: CIOMS. Important research methods in a clinical trial include randomization,101Berkman et al., op. cit. note 13, p. 286. blinding, and controls.102Brody, B. A. (1997). When are placebo‐controlled trials no longer appropriate? *Controlled Clinical Trials, 18*, 602–612, p. 605. These elements of research design guarantee that the research results in statistically significant data that can be used to attain that anticipated future benefit of improving treatment prospects for preterms. Without such data, there can be no guarantee that AWT produces any benefit, and it would be unethical to continue to expose future subjects to the associated risks. However, these research methods, in particular randomization, invoke ethical questions. Because the AWT research trial would involve comparing the new technology against the existing technology, prospective subjects would be randomized to receive *either* NIC *or* AW care. Only making this direct comparison in outcomes under controlled conditions would allow researchers to conclude whether AWT is/is not effective.

The concern with randomization is that some subjects are ‘disadvantaged’ because they receive an inferior treatment (whether this is NIC or AWT) in a research trial. There is broad consensus, according to Freedman, that to be ethical, clinical research must begin with an ‘honest null hypothesis’, meaning that there is a ‘state of genuine uncertainty regarding the comparative merits of treatments A and B for population P’.103Freedman, B. (1987). Equipoise and the ethics of clinical research. *New England Journal of Medicine, 317*, 141–145, p. 141. Uncertainty means that there is insufficient evidence to establish that treatment A *is* better than treatment B (or vice versa) for that population, and thus any individual subject is not disadvantaged by receiving one treatment rather than the other. This uncertainty, where there exists ‘an honest, professional disagreement among expert clinicians about the preferred treatment’ is known as clinical equipoise.104Ibid: p. 141. Depending on the clinical population identified as the potential subjects of an AWT trial, clinical equipoise could offer some resolution to the discomfort surrounding randomization. The best evidence available regarding the efficacy of AWT at the time of initial trials would be animal studies, and outcomes for human subjects would be innately uncertain. If we accept the importance of clinical equipoise as an ethical constraint,105For arguments defending the concept of clinical equipoise, see Hey, S., London, A. J., & Weijer, C. (2017). Is the concept of clinical equipoise still relevant to research? *British Medical Journal, 359*, j5787. the treatment for comparison must also be uncertain. Outcomes in NIC markedly improve with gestational age. Identifying the requisite threshold of uncertainty using prediction models (likely based on prospects for a good outcome) would need to be carefully determined. What chance of survival in NIC would be deemed comparatively uncertain? In medical practice, the viability threshold is defined as the gestational age at which the chance of survival with NIC is 50%106Glass et al., op. cit. note 34, p. 1338. (usually this point is reached at 24 weeks gestation).107Ibid. If we accept that a 50% chance of survival is a useful threshold, it is the point at which treatment in NIC is usually deemed appropriate, preterms *below* this point with a lesser chance of surviving might satisfy a claim that there is insufficient evidence to suppose that AWT would result in a worse outcome. The point of sufficient uncertainty, however, is hard to quantify. Identifying when the likelihood of survival (based on prediction models) is comparatively uncertain to the unknowns in AWT is, ultimately, a subjective matter. It might be argued that a 25% chance of survival in NIC is not comparatively uncertain to AWT, and it equally might be argued that it is. To further complicate things, the analysis so far is dependent on the assumption that the only relevant consideration in identifying a null hypothesis is survival. Long‐term morbidity is also considered an important factor in determining the appropriateness of providing or withdrawing current NIC treatment. How might we determine the point at which a chance of certain morbidities is deemed sufficient to gamble on the uncertainty in AWT? Moreover, what kind of complications would be relevant? Gestational age probably would still be an important factor in this determination, because morbidity rates, while still high among all NIC patients, are greater at or below the viability threshold. The critical point is that with these necessary research methods, it is more justifiable to enrol ‘almost viable’ rather than ‘just viable’ subjects in the study. It is easier to apply the concept of clinical equipoise to this group because they are less likely to be exposed to more risk in the study than they would be receiving standard care.

It is expressly within the remit of an IRB to consider the inclusion criteria of a proposed clinical trial.108U.S. Food & Drug Administration (2009*). FDA 101: Clinical Trials and Institutional Review Boards.* Retrieved from: https://www.fda.gov/consumers/consumer-updates/fda-101-clinical-trials-and-institutional-review-boards. Identifying the ‘almost viable’ (as opposed to the ‘just viable’ preterm) as the target population of clinical trials seems prima facie more justifiable. The OHRP Regulations also direct towards this conclusion, because they require that research holds out the ‘prospect of enhancing the probability of survival of the [developing human entity of uncertain viability]… and any risk is the *least possible* for achieving that objective’ [emphasis added].109HHS Regulations, op. cit. note 88, §46.205 (1) (ii). However, it is not uncommon for NIC to be withheld or withdrawn from this population because treatment (ventilation, nasogastric feeding etc.) is often thought to be too burdensome for extreme preterms with limited prospects for improvement.110Lantos, J. D., & Meadow, W. L. (2006) *Neonatal bioethics: The moral challenges of bioethical innovation.* Baltimore: John Hopkins University Press, pp. 109–110. ‘Almost viable’ subjects may be inherently more vulnerable because of the greater likelihood of treatment being futile, and therefore potentially an unethical burden.111Ethical arguments for the withdrawal of NIC treatment thought to be futile, and therefore only likely to prolong suffering, have found strong support in the law. Courts (in England and Wales) have overwhelmingly supported the conclusion that withdrawing treatment in these circumstances is in a preterm’s best interests: Bhatia, N. (2015) *Critically impaired infants and end of life decision making: Resource allocation and difficult decisions.* Abingdon, U.K.: Routledge, pp. 41. How we might balance the researchers’ interest in gathering useful data against some of the ethical considerations in burdensome treatment, in both arms of the trial, would need to be carefully navigated. Moreover, to what extent is opportunity for survival worth the potential for suffering in that process (by subjects in both arms of an AWT trail)? Regulatory requirements impose obligations on researchers to minimize risks and maximize potential benefits for subjects.112Mason & Laurie, op. cit. note 72, p. 659. We would need to carefully determine the limits of harm exposure in the experiment. Context is crucial. Lantos explains that in the setting of NIC, standard care is already predictably associated with serious risk of morbidity and mortality.113Lantos, J. D. (2018) Neonatal research ethics after SUPPORT. *Seminars in Fetal & Neonatal Medicine, 23*, 68–74, p. 71. We must be careful, therefore, in not judging the study based on risk in its entirety, but by considering whether there is any attributable risk, which Lantos defines as the risk ‘attributed to study participation as opposed to being attributed to the clinical condition of… [the subject] and the known risk of conventional treatments’.114Ibid: p. 70. Those subjects randomly assigned to NIC in a potential AWT trial would not be exposed to any attributable risk. Presumably, if the parent(s) were willing to consent to the study, they would also be consenting to standard care in NIC. The group receiving AWT is potentially of more significance, but given the high risk of death/injury in ‘almost viable’ subjects, attributable risk is likely to be minimal. There should also be a consideration of safeguards to ensure the safety of research participants. For example, in a recent study evaluating oxygen saturation targets in preterm neonates, a data safety monitoring board was established that continuously compared the incidence rates of serious adverse events between arms of the study.115Ibid: p. 70. Thus, if a statistically significant trend emerges demonstrating that either arm of the trial is resulting in more frequent adverse results, the study can be stopped.

Finally, we must consider how consent for AWT research should be obtained. In neonatal/foetal research, informed consent must be obtained from the parent(s) of the subject.116The HHS Regulations, op. cit. note 88, §46.205 (2) stipulate that effective consent to research on a neonate must be provided by either parent, and, in the case of non‐viable neonates, §46.205 (c) (5) is clear that both parents must provide effective consent unless one is unavailable. §46.204(e) is explicit that in the case of research on foetuses where there is only the prospect of direct benefit to the foetus (and not the pregnant person), the effective consent of both parents must be sought unless the father is unavailable to provide it. They will only be able to decide to permit participation in a trial if they are provided with adequate information to make an informed decision. The law (of multiple jurisdictions) requires an even greater degree of risk disclosure to parent(s) for research as opposed to treatment.117Biggs, H. (2009). *Healthcare research ethics and law: regulation, review and responsibility*. London, U.K.: Routledge‐Cavendish, pp. 5–7, 82. Requiring a more stringent consent, involving an understanding of the purpose of the research, any potential risks or benefits, and the alternatives involved,118Mason & Laurie, op. cit. note 72, p. 668. is an important mechanism to ensure that parent(s) are not coerced. Principal investigators should be able to clearly explain to participants’ parent(s) what the standard course of care would be, and how being in the trial would be different. In the case of AWT, parent(s) should be counselled about the options regarding not enrolling in the study, and the potential harms and benefits of enrolling (including randomization). Some scholars have expressed concern about parents’ ability to meaningfully consent to research, because of the emotional challenges involved in decisions about giving their offspring a ‘chance of life’.119Harrison, H. (2008) The offer they can’t refuse: Parents and perinatal treatment decisions. *Seminars in Fetal and Neonatal Medicine, 13*(5), 329–334. Specific aspects of the process, for example randomization, can also be distressing to comprehend.120Kodish, E., Eder, M., & Noll, R. B. (2004) Communication of randomization in childhood leukemia trials. *Journal of the American Medical Association, 291*(4), 470–475, p. 472. Uncertainty is hard to communicate. The explanation that this process may be unlikely to benefit this subject, but may be able to help preterms in the future will be a difficult conversation for researchers to navigate. The emotional impact on parent(s) is a factor that IRBs must carefully consider.121I am grateful to one of my anonymous reviewers for raising this point.


One of the biggest problems with research is minimizing the effect of ‘therapeutic misconception’ when individuals presume that the experiment in which they are participating (or agreeing their preterm should participate in) is medical treatment.122Appelbaum, P. S., Roth, L. H., Lidz, C. W., Benson, P., & Winslade, W. (1987). False hopes and best data: Consent to research and the therapeutic misconception. *Hastings Center Report, 17*(2), 20–24, p. 20. Parent(s) most likely to agree to experimental AWT are those who believe that the experimental technology provides the best possible chance at life, or potentially of life without complications following NIC. Experimental AWT *may* benefit some of the first preterms that are subjected to it. There is, however, the danger that parents may not appreciate the real risk that experimental AWT may not work, or may actively cause harm. Such expectations about new approaches are best managed in the context of a research trial because of a shift in perception. There is increased awareness that the purpose of receiving the treatment is to test the hypothesis about a new approach, with the understanding that good results are not likely, or not without risk, or that standard care is received because of randomization. If the consent process if designed carefully, it should meaningfully address therapeutic misconception. Discussion about treatment in the distressing circumstances that a pregnancy is going to end/has ended prematurely will always be difficult. Lantos and Feudtner emphasize that grappling with painful decisions, and the details in the process, will be especially hard for parent(s) when time is limited—sometimes they may have only minutes123Lantos, J. D,. & Feudtner, C. (2015) SUPPORT and the ethics of study implementation. *Hastings Centre Report*, *45*(1), 30–40, p. 38.—and when they feel pressured by a sense of emergency. They argue, however, that meaningful consent can still be carefully procured if processes are simplified and parent(s) given ‘key information honestly and straightforwardly’.124Ibid: p. 38. Entirely how we determine a sufficient consent process for an AWT trial must be the subject of further discussion.

## 5 CONCLUSION

Two research teams have claimed proof of principle for AWT. It is plausible that soon there could be calls to test their devices, which might drastically improve the prospects for preterms, on humans. It is necessary, therefore, to consider how and when we should use experimental AWT on humans. Little attention has been paid to the ethical issues in AW research. It is often assumed that AWT constitutes an innovative, beneficial treatment, and thus would emerge almost ‘by accident’. The tendency to conceptualize experimental uses of AWT as innovative treatment—as extensions of current NIC—and therefore justifiable in the best interests of preterms is flawed.

In this paper, I demonstrated that AWT is medical research. AWT is conceptually distinct from NIC, and it is unknown if it will produce desirable outcomes. Experimental AWT is not ipso facto in any individual preterm’s best interests. Clinical trials will be a necessary part of the clinical translation of these devices because of regulatory processes. Clinical trials are thought to ensure protection for research subjects, to produce reliable, generalizable knowledge, and to better manage therapeutic misconceptions. However, these benefits are contingent upon the conditions of the research trials. Moreover, clinical trials would only be justifiable under particular conditions. In this paper, I highlighted some of the important ethical questions that would need to be considered in designing research trials of AWT devices. I considered the appropriate clinical population for such a trial, the ethical issues in research methods such as randomization, and the importance of carefully addressing consent and therapeutic misconception. These identified issues remain in need of further scrutiny, but it is hoped that this paper has started the necessary ethico‐legal conversation about experimental AWT. It is common in the literature for the development and use of AWT in humans to be discussed as a foregone conclusion. However, we should be careful to avoid focusing on the potential implications of AWT while neglecting issues in the development of the technology.

I am grateful to Dr Alexandra Mullock, Professor Bernard Dickens, Professor Rebecca Bennett and fellow students at the Centre for Social Ethics and Policy for their helpful comments on earlier drafts of this paper.

## CONFLICT OF INTEREST

The author declares no conflict of interest.

